# Profile of Hemoglobin D (HbD) Disease in Eastern Uttar Pradesh: A Single-Center Experience

**DOI:** 10.7759/cureus.30782

**Published:** 2022-10-27

**Authors:** Vineeta Gupta, Priyanka Aggarwal

**Affiliations:** 1 Pediatrics, Institute of Medical Sciences, Varanasi, IND

**Keywords:** hydroxyurea, beta thalassemia trait, sickle cell trait, anemia, hbd, hemoglobinopathy

## Abstract

Hemoglobin D (HbD) disease was identified in 31 samples from 15 families out of the 2560 samples (1.20%) analyzed for variant Hbs. There were five patients with HbSD disease, three with HbDβ disease, and the remaining 23 were HbD trait. Patients with HbSD disease had a variable clinical presentation with a pair of siblings being transfusion dependent although the age of first blood transfusion was different in the two patients. The one with high HbF started transfusions much later. None of them had symptoms related to sickling. Patients with HbDβ also had a variable presentation with only one of them being transfusion-dependent. All patients with HbSD and HbDβ disease were started on hydroxyurea. Persons with HbD trait were asymptomatic with half of them having normal Hb. The remaining half had mild microcytic hypochromic anemia. All the families with HbD disease were natives of this region and not migrants from other states. Although HbD disease has not been reported from this region in previous studies, clinicians need to be aware of this entity as it can give rise to symptomatic disease in some cases if associated with beta-thalassemia or sickle cell trait.

## Introduction

Hemoglobin D (HbD)-Punjab is a common Hb variant distributed worldwide and follows HbS and HbC in frequency. It results from glutamate to glutamine substitution at codon 121 of the beta chain whereas HbS and HbC result from valine for glutamic acid substitution and replacement of glutamate by lysine at position 6 of the beta chain respectively. HbD disease is common in countries such as Italy, Belgium, Austria, and Turkey. In Turkey, it is the most common Hb variant in one of the provinces comprising 57.9% of abnormal Hbs followed by HbS with 21.9%. Similarly, in China also it is the most common Hb variant in Xinjiang province comprising 55.6% of variant Hbs [1]. In India, it is prevalent in Punjab which gives it the name HbD-Punjab, and northwestern India with an estimated frequency of 2.0%. Studies from other parts of the country have reported a frequency ranging from 0.06% to 0.2% in the samples evaluated for hemoglobinopathies [2-4].

Both heterozygous and homozygous forms of HbD-Punjab are largely asymptomatic with some of them presenting with microcytic hypochromic anemia. But a combination with other Hb variants can give rise to a moderate to severe disease. There are very few studies from Eastern Uttar Pradesh profiling the frequency of Hb variants in this part of the country and none on the profile of HbD disease [5-6]. Therefore, the presentation of the disease is not known in this area. We report our experience of the clinical spectrum of HbD disease diagnosed in a tertiary care university hospital in Eastern Uttar Pradesh.

## Materials and methods

A total of 2560 blood samples from patients under evaluation for anemia, those undergoing voluntary carrier testing, and family screening were analyzed for thalassemia and other hemoglobin variants. Detailed history including family, clinical, and social history was taken in all the cases. Demographic details included the area of residence, ethnicity, caste, and history of migration from other states. Clinical details included age at onset of symptoms, age at diagnosis, sex, presenting complaints, any episodes of pain or history suggestive of vaso-occlusive crisis (VOC), number of blood transfusions, clinical complaints, treatment received, and duration of the last follow-up. A thorough physical examination was carried out to evaluate for hemolytic facies, pallor, icterus, and splenomegaly.

Blood samples were collected for complete blood cell count including red cell indices. A peripheral smear was prepared for each patient, stained with Leishman stain, and examined microscopically for red cell morphology. Reticulocyte count was performed by staining with methylene blue and a sickling test was performed with 2% sodium metabisulfite. Patients with a history of blood transfusion within the last four weeks were excluded.

Quantitation of HbA, HbS, HbF, and HbD was carried out with high performance liquid chromatography (HPLC) on Biorad Variant II using beta thalassemia short program (Bio-Rad Laboratories, CA, USA) which has been found to be reliable tool for diagnosis of Hb variants. Hbs were identified by their retention time (RT) and quantified by computing the area under the corresponding peak in the elution profile. Genetic analysis with amplification refractory mutation system-polymerase chain reaction (ARMS-PCR) and/or next-generation sequencing was carried out to identify the specific mutations in those found to have Hb variants. Those with HbD disease on high performance liquid chromatography (HPLC) were subjected to next-generation sequencing. Patients with HbSD disease had two mutations: c.364G>C (HbD) and c.20A>T (HbS) and patients with HbDβ had a combination of c.364G>C (HbD) and c.92+5G>C (IVS1-5).

Patients were regularly followed up in the Centre for Thalassemia and hemoglobinopathies. Their Hb, frequency of blood transfusions, episodes of pain crisis, and response to treatment were carefully monitored.

## Results

Hemoglobin D disease was identified in 31 samples (1.20%) from 15 families. There were five patients with HbSD disease, three with HbDβ, and 23 were with HbD trait. Patients with HbSD disease belonged to four families where two were siblings from one family. All the families were residents of Sonebhadra district which has a sizeable tribal population. One family each belonged to the upper caste, scheduled caste, scheduled tribe, and Muslim community. The siblings were transfusion-dependent. The age at first transfusion in the siblings was two years (HbF 16.1%, HbD 23.0%) and six years (HbF 30.4%, HbD 19.3%) respectively. A 12-year-old (HbF 15.0%, HbD 41.8%) and a 4-year-old (HbF 25.5%, HbD 42.8%) are transfusion independent till now. Another two-year-old with HbSD disease was lost to follow-up after one year. The mean mean corpuscular volume (MCV) and mean corpuscular hemoglobin (MCH) of the patients with HbSD disease were 80.9±10.7 fl and 32.6±2.5 pg, respectively. All the patients with HbSD disease were started on hydroxyurea at a dose of 10-15 mg/kg/day. None of the patients have complained of symptoms related to sickling at any time. The clinical profile of the patients has been presented in Table 1.

**Table 1:** Clinical profile of patients with HbD disease. Hb, hemoglobin

S. No	Age	Sex	HbA (%)	HbA2 (%)	HbF (%)	HbD (%)	HbS (%)	Diagnosis
1.	37	F	52.0	1.5	0.2	38.4	-	HbD trait
2.	13	F	7.6	2.5	5.2	81.3	-	HbDβ thalassemia
3.	9	M	8.4	2.6	4.8	80.6	-	HbDβ thalassemia
4.	28	F	51.2	0.9	0.2	36.9	-	HbD trait
5.	3	F	41.8	0.7	16.1	23.0	11.7	HbSD disease
6.	8	M	2.9	0.5	30.4	43.5	19.3	HbSD disease
7.	3	M	51.2	0.5	1	35.8	-	HbD trait
8.	26	F	52.2	0.6	0.8	36.0	-	HbD trait
9.	12	F	55.5	0.3	0.3	32.6	-	HbD trait
10.	9	M	50.4	0.6	0.2	37.4	-	HbD trait
11.	9	M	56.5	0.8	0.7	34.8	-	HbD trait
12.	34	M	51.0	0.4	0.7	36.8	-	HbD trait
13.	12	M	3.6	2.4	15.0	41.8	32.8	HbSD disease
14.	37	M	49.7	2	0.3	38.1	-	HbD trait
15.	25	F	51.2	1.3	0.3	37	-	HbD trait
16.	40	M	50.1	1.5	0.2	37.4	-	HbD trait
17.	5	M	50.9	1.7	0.9	36.3	-	HbD trait
18.	22	F	50.7	2	0.5	37.2	-	HbD trait
19.	2	M	50.2	1.6	13.6	15.5	7.2	HbSD disease
20.	13	F	6.2	2.1	0.6	83.7	-	HbDβ thalassemia
21.	38	F	54.3	0.7	0.6	36.2	-	HbD trait
22.	1	F	58.4	1.2	2.2	31.6	-	HbD trait
23.	22	F	52.6	0.9	0.6	35.4	-	HbD trait
24.	4	M	2.2	2.1	25.5	42.8	25.9	HbSD disease
25.	46	M	53.9	1.9	0.2	33	-	HbD trait
26.	17	F	55.1	1.4	0.5	33.2	-	HbD trait
27.	24	M	51.1	1.1	1.6	36.9	-	HbD trait
28.	19	M	51.3	1.1	0.3	36.1	-	HbD trait
29.	1.5	F	61.6	1.5	1.1	24.5	-	HbD trait
30.	28	F	60	2	0.7	27.9	-	HbD trait
31.	24	F	53	1.2	0.6	36	-	HbD trait

Patients with HbDβ belonged to two families where two were siblings from one family. Both families belonged to the backward castes of Varanasi district. One of the siblings was transfusion dependent whereas the other 13-year-old was non-transfusion dependent (HbF 5.2%, HbD 81.3%). The third child, a 13-year-old is also non-transfusion dependent (HbF 0.6%, HbD 83.7%). Both non-transfusion patients are receiving hydroxyurea and maintaining Hb of 7.5-8.0 g/dL.

Persons with HbD trait had a mean Hb of 11.5 ± 2.6 g/dL. Approximately half of the patients had mild microcytic hypochromic anemia with MCV 71.6 ± 10.7 fl and MCH 24.2 ± 4.6 pg. They were asymptomatic and were diagnosed during carrier testing or family screening. They were counseled regarding their carrier status and no specific treatment was given.

## Discussion

The HbD-Punjab and HbD-Los Angeles are the same Hb variants that were discovered in a multi-ethnic family of Indian origin living in the United States. Glutamine substitutes in glutamic acid at position 121 of the globin chain. HbD has similar electrophoretic mobility as HbS in alkaline pH whereas it has mobility similar to HbA in acidic pH. On HPLC, it has an RT of 3.90-4.30 min and elutes after HbA2 (RT 3.30-3.90 min) but before HbS (RT 4.30-4.70 min). HbD disease is prevalent in Punjab and northwestern India but has been reported in other areas also. There are some studies from eastern Uttar Pradesh profiling the pattern of hemoglobinopathies but none of them have reported HbD disease [5-6]. In a study conducted on individuals residing in Lucknow, Uttar Pradesh, a prevalence of 1.5% for the HbD trait was found [7]. The prevalence was found to be 3.1% in the Khatri community and 0.5% in other Hindus. In our study, we did not come across such distribution. The cases were distributed in all castes native to this region. Patients with HbSD disease were from a particular district that has a high prevalence of sickle cell anemia.

HbD-Punjab can be inherited as a homozygous disease, heterozygous trait, or in a compound heterozygous state with other Hb variants. Homozygous and heterozygous HbD cases may be completely asymptomatic or may have mild microcytic hypochromic anemia. However, a combination of the HbD trait with the HbS trait or beta thalassemia trait can give rise to symptomatic patients. Patients with HbSD disease may have varied clinical presentations ranging from mild symptoms to transfusion dependence and/or sickling symptoms due to the facilitation of the polymerization of HbS by the β glutamine residue of HbD-Punjab.

In our study, patients with HbSD disease had a variable presentation with two of the patients being transfusion dependent although their age at first blood transfusion was different. We ascribed it to their HbF level which was high in the patient who became transfusion-dependent late [8]. Interestingly, none of the patients had symptoms related to sickling. In a study on HbSD disease which included 10 patients, all the patients had moderate to severe anemia and VOC and acute chest syndrome were observed in approximately half of the patients. Improvement in hemoglobin and reduction in painful episodes was observed with hydroxyurea treatment [9]. Similar observations were made in another study comparing the phenotypic expression of HbSS and HbSD which stated that fetal Hb and alpha thalassemia modulate the phenotypic expression of HbSD-Punjab [10]. HbF had a protective effect on the frequency of VOC in HbSD. However, in our study association with alpha thalassemia could not be evaluated due to financial constraints.

Patients with HbDβ thalassemia also had a variable presentation with one of the patients being transfusion-dependent while the other two were non-transfusion-dependent. They also had normal HbA2 levels (our laboratory reference value for HbA2: 2.5%-3.5%) with raised HbF for age. One of the patients with a low HbA2 level of 2.1% had concomitant iron deficiency anemia. Again an association with alpha thalassemia could not be studied in patients with HbDβ disease which could have modulated the clinical phenotype. The variable clinical presentation of HbDβ disease has been reported in another study also where of the three patients with HbDβ disease, only one had a diagnosis made on clinical symptoms. The other two patients were found in routine prenatal screening which further demonstrates the variability in the clinical picture of coinheritance of HbD-Punjab with beta-thalassemia [11].

Approximately half of the persons with HbD-Punjab trait had normal hemoglobin with normal red cell indices while the remaining had mild to moderate microcytic hypochromic anemia. This is similar to the observation of other studies.

## Conclusions

In conclusion, the presence of HbD disease was observed in the native population of eastern Uttar Pradesh. Clinicians need to be aware of the condition as the HbD trait is asymptomatic but a combination with HbS or beta thalassemia trait can give rise to severe symptomatic disease.
